# Fulfilling the Promise of Personalized Medicine? Systematic Review and Field Synopsis of Pharmacogenetic Studies

**DOI:** 10.1371/journal.pone.0007960

**Published:** 2009-12-02

**Authors:** Michael V. Holmes, Tina Shah, Christine Vickery, Liam Smeeth, Aroon D. Hingorani, Juan P. Casas

**Affiliations:** 1 Centre for Clinical Pharmacology, University College London, London, United Kingdom; 2 Department of Epidemiology and Population Health, London School of Hygiene and Tropical Medicine, London, United Kingdom; 3 Department of Epidemiology, University College London, London, United Kingdom; University of Maryland School of Pharmacy, United States of America

## Abstract

**Background:**

Studies of the genetic basis of drug response could help clarify mechanisms of drug action/metabolism, and facilitate development of genotype-based predictive tests of efficacy or toxicity (pharmacogenetics).

**Objectives:**

We conducted a systematic review and field synopsis of pharmacogenetic studies to quantify the scope and quality of available evidence in this field in order to inform future research.

**Data Sources:**

Original research articles were identified in Medline, reference lists from 24 meta-analyses/systematic reviews/review articles and U.S. Food and Drug Administration website of approved pharmacogenetic tests.

**Study Eligibility Criteria, Participants, and Intervention Criteria:**

We included any study in which either intended or adverse response to drug therapy was examined in relation to genetic variation in the germline or cancer cells in humans.

**Study Appraisal and Synthesis Methods:**

Study characteristics and data reported in abstracts were recorded. We further analysed full text from a random 10% subset of articles spanning the different subclasses of study.

**Results:**

From 102,264 Medline hits and 1,641 articles from other sources, we identified 1,668 primary research articles (1987 to 2007, inclusive). A high proportion of remaining articles were reviews/commentaries (ratio of reviews to primary research approximately 25∶1). The majority of studies (81.8%) were set in Europe and North America focussing on cancer, cardiovascular disease and neurology/psychiatry. There was predominantly a candidate gene approach using common alleles, which despite small sample sizes (median 93 [IQR 40–222]) with no trend to an increase over time, generated a high proportion (74.5%) of nominally significant (p<0.05) reported associations suggesting the possibility of significance-chasing bias. Despite 136 examples of gene/drug interventions being the subject of ≥4 studies, only 31 meta-analyses were identified. The majority (69.4%) of end-points were continuous and likely surrogate rather than hard (binary) clinical end-points.

**Conclusions and Implications of Key Findings:**

The high expectation but limited translation of pharmacogenetic research thus far may be explained by the preponderance of reviews over primary research, small sample sizes, a mainly candidate gene approach, surrogate markers, an excess of nominally positive to truly positive associations and paucity of meta-analyses. Recommendations based on these findings should inform future study design to help realise the goal of personalised medicines.

**Systematic Review Registration Number:**

Not Registered

## Introduction

Individual differences in drug efficacy, or susceptibility to adverse effects, collectively make an important contribution to the burden of ill-health [1], [2]. Studying the genetic basis could reduce this by clarifying pathways and mechanisms of drug action or metabolism to inform drug development, and by the development of genotype-based predictive tests of efficacy or toxicity (pharmacogenetics).

As with research in common disease susceptibility, the path to translation involves a two stage process that first requires the reliable identification of the genetic loci involved, and then research into the healthcare applications of this knowledge, which includes critical appraisal of the performance of genotype as a predictive test. While the extent of the clinical impact of research in both areas is uncertain, the reliable identification of loci involved in drug response (pharmacogenetics) appears to be less advanced than the identification of susceptibility loci for common disease [3]. After more than two decades of research, a continuing expansion in the range and depth of available drug therapies, and the continued promise of ‘personalized medicine,’[4], [5], [6], [7], [8], [9] only four pharmacogenetic tests were mandated as part of the FDA drug approval pre-July 2009, [10] while for another 10 tests recommended by the FDA, clinical utility is not universally agreed [11], [12], [13]. Understanding the reasons for the blocks in development of personalised medicines could help improve efficiency of future research.

Systematic reviews and field synopses previously exposed the obstacles to progress in complex disease genetics. These included: a focus on candidate genes rather than genome-wide analysis; inadequate sample size; suboptimal capture of genetic variation; and significance chasing and reporting bias; all of which led to a failure to replicate and validate genetic associations [14], [15], [16]. These overviews [17], [18], [19] were followed by improvements in research design which made an important contribution to the recent success in the identification in genes for common disease [20]. These considerations and the absence of a prior systematic, quantitative overview of pharmacogenetic research was the motivation for the current study.

## Methods

We followed PRISMA 2009 guidelines [21].

### Search Strategy

We identified pharmacogenetic studies using a carefully designed search strategy. We searched articles indexed in Medline using the Medical Subject Heading (MeSH) or full text terms (“Genetic Variation”[MeSH] or “Genotype”[MeSH] or “Genes”[MeSH] or genotype* or polymorphism* or allele* or mutation*) and (“Treatment Outcome”[MeSH] or “Therapeutics”[MeSH] or “adverse effects”[Subheading] or “Pharmacogenetics”[MeSH] or “Toxicogenetics”[MeSH] or pharmacogenomic* or pharmacogenetic* or toxicogenetic* or therapeutic* or intervention* or treatment*) from inception up to 01-01-2008. The search was initially restricted to Human studies and subsequently to Clinical Trials, Meta-Analyses, Practice Guidelines, and Randomized Controlled Trials using the Medline filters and by doing so excluded Editorials, Reviews and Letters. We supplemented the search with relevant references indexed in 12 meta-analyses and 12 review articles (spanning most disease categories). The FDA “Table of Valid Genomic Biomarkers in the Context of Approved Drug Labels”[10] was also cross-referenced and to identify potentially missing meta-analyses, the ten most frequently studied genes in each category (germ-line [kinetic/dynamic] and somatic) were individually searched in Medline (none extra was found). Furthermore, as some meta-analyses are indexed in Medline as reviews and thus had the potential to be excluded during the initial search, we repeated our Medline search selecting only meta-analyses.

To be eligible for inclusion, studies had to satisfy our definition of a pharmacogenetic study: a study in which the response (intended outcome/adverse reaction) to drug therapy was examined in relation to genetic variation (germline/somatic) in humans. It was mandatory that participants be genotyped (studies using phenotype as a surrogate of genetic variation were excluded) and that >1 allelic variations at a gene were analysed (in order to compare differing alleles on response to treatment). All abstracts from the Medline search were screened to determine if they fulfilled the inclusion criteria by MH, aided by CV. Two authors blindly assessed a random subset of abstracts to corroborate inclusion and exclusion (JPC, AH). One hundred and sixty-one articles were chosen at random (∼16 papers/year from 1998–2007 inclusive) and full texts were scrutinized in more detail.

### Data Extraction

The following were extracted and recorded from the abstracts of included articles: year of publication; first author; journal name; continent of correspondence; language of publication; disease category; study design; gene(s) studied and whether variation was in somatic (cancer) cells or in the germline and, if germline, whether related to drug absorption/distribution/metabolism/elimination (pharmacokinetic) or the drug target (pharmacodynamic) and whether the study was primarily set up to investigate the pharmacogenetic end-point. We also extracted information on: the primary outcome including whether this was the intended or an adverse effect of the drug; the number and magnitude of reported p values in each study (categorized as only non-significant p values [p>0.05], only significant p values [p≤0.05] and mixed [p values both ≤ and >0.05]); specific drugs, further classified according to the British National Formulary coding (http://www.bnf.org accessed 2009 November 10, archived URL http://www.webcitation.org/5lBYIOLVR) and the 2006 impact factor of the publication (derived from Journal Citation Reports ® ISI Web of Knowledge^SM^ (http://www.isiwebofknowledge.com accessed 2009 November 10, archived URL http://www.webcitation.org/5lBTT863z) grouped into 0 to 4.99, 5–9.99 and ≥10. From the 161 full-text articles, data were also extracted on: (i) genes and alleles investigated, including the mean allele frequency (MAF); (ii) outcomes, classified according to their clinical end-point into binary and continuous; (iii) the number of analyses and p values reported from gene-drug interactions.

### Definition of Disease Category

Disease categories were organ-specific with the exception of (i) cancer, which encompassed any body site in which there was neoplasia, and (ii) anti-coagulation, classified as ‘cardiovascular’. The cardiovascular disease category also included acute myocardial infarction and peripheral vascular disease; neurology/psychiatry included stroke, psychosis, and depression; endocrine disease included diabetes and hyperlipidaemia (where the outcome assessed was a change in lipid level and not the effect on cardiovascular end-points).

### Gene Nomenclature and Classification

Genes were named according to HUGO (HUman Genome Organisation) Gene Nomenclature Committee (HGNC, Wellcome Trust; http://www.genenames.org accessed 2009 November 10, archived URL http://www.webcitation.org/5lBCXvH6E). The classification of genes into dynamic or kinetic was checked with the Pharmacogenomics Knowledge database (PharmGKB; http://www.pharmgkb.org accessed 2009 November 10, archived URL http://www.webcitation.org/5lBChBcLk). Where it was not possible to precisely classify the specific gene according to HUGO nomenclature, an asterisk was placed after the initial characters (e.g. HTR* denotes serotonin receptor genes, of which *HTR1B* and *HTR2A* are specific examples).

### Outcomes Recorded

A study in which the outcome investigated was the desired effect of the drug (e.g. pH lowering from use of a proton pump inhibitor) was defined as ‘intended effect’; one in which the outcome was adverse was classified as an ‘adverse effect’ (this encompassed both hypersensitivity and dose-dependent adverse reactions).

For the 161 full-text papers, outcomes were classified as binary or continuous: examples of binary were death, disease recurrence, or an episode of bleeding; examples of continuous were changes in the plasma levels of a drug, gastric pH or international normalised ratio (INR, e.g. for the monitoring of warfarin anticoagulation).

### Continent of Correspondence

The continent of correspondence was determined from the Medline citation and used as a surrogate marker for the geographic location of the study.

### Study Design

The study design was categorized as: (i) prospective (including randomized clinical trials), (ii) case-control, (iii) cross-sectional, or (iv) meta-analysis.

### Primary/Secondary Pharmacogenetic Study

A primary pharmacogenetic study was defined as one in which the title of the study or the stated aims or purpose within the text of the abstract indicated that the primary intention of the study was to investigate the effect of genetic variation on drug response. If not explicitly stated, the study was classified as a secondary pharmacogenetic study.

### Exclusions

We excluded the following as ‘drug’ treatments: ionizing radiation, surgical procedures, non-drug-eluting stents, bone marrow transplantation, tobacco, alcohol, environmental agents or pollutants (e.g. lead), herbal remedies, dietary or lifestyle interventions including acupuncture, massage, counseling, or exercise.

### U.S. Food and Drug Administration (FDA) Guidelines

We analysed the evidence-base behind the FDA list of approved pharmacogenetic tests (pre-July 2009) [10]. The articles cited in support of FDA labeling as ‘test required’ or ‘test recommended’ were reviewed (Document S1). Tests (gene and drug pairs) were cross-referenced with the generated database. FDA recommendations were contrasted with guidelines from authoritative medical bodies.

### Statistical Analysis

Statistical analyses were performed using SPSS for Windows version 17.0 and Stata 10. A value of p≤0.001 was taken as significant. Frequency distributions were analysed for normality by 2-tailed Chi-Square. Impact factors were ranked by Mann-Whitney U. Sample sizes were converted into logarithmic (log_e_) values and means compared with unpaired student's t.

## Results

### Articles Retrieved

**Figure 1:**
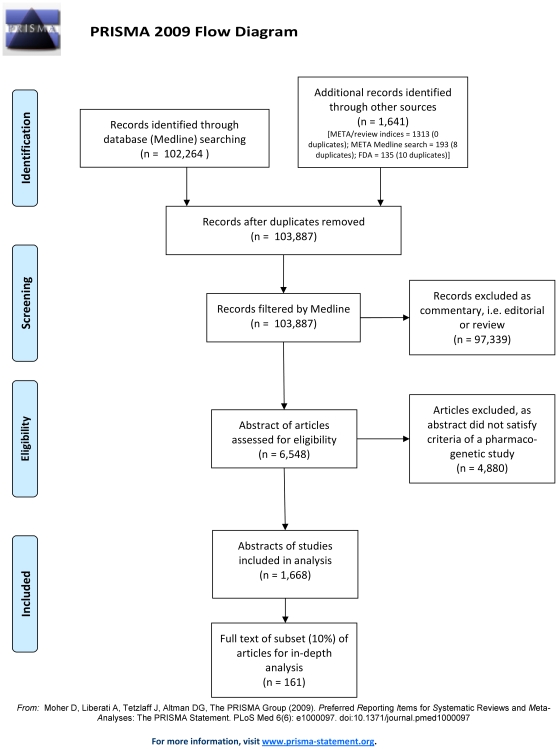
Flow chart of methodology for identifying pharmacogenetic studies in the systematic review. From PRISMA 2009 guidelines [21].

**Figure 2:**
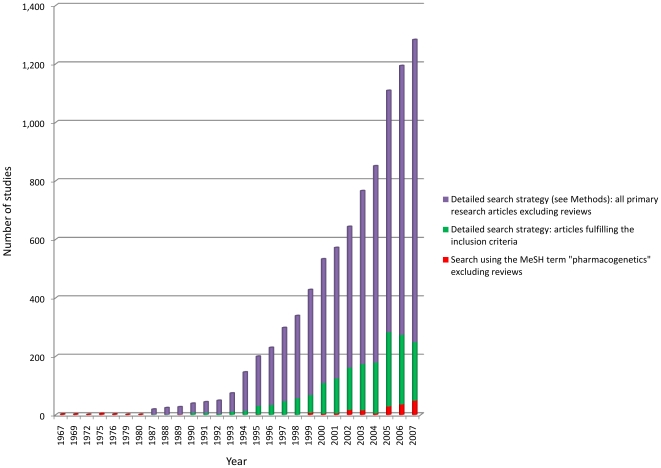
Growth in publications in the field of pharmacogenetics from 1967–2007 (inclusive). Our detailed search strategy incorporating both Medical Subject Headings (MeSH) and free-text terms (filtered for Humans and excluding Reviews/Editorials) identified 6,548 original articles (purple bars) of which 1,668 fulfilled the inclusion criteria (green bars). By contrast the total number of articles obtained based on a search using the MeSH term “pharmacogenetics” (including reviews and editorials) was 4,674, of which only 183 were original articles (red bars), indicating a ratio of approximately 1∶25 of original research to commentary/review.

A sensitive, non-specific search strategy in Medline (see Methods) yielded 102,264 articles (Figure 1) with an additional 1,641 articles identified from other sources. 97,339 (94%) articles were annotated as reviews, editorials or letters rather than primary research, and were excluded. Of the 6,548 remaining articles, a total of 1,668 (1.6% of studies from the initial search) reported original research that fulfilled all our inclusion criteria. A much less sensitive search strategy utilising the MeSH term “pharmacogenetics” retrieved only 4674 articles, of which 183 (4%) were indexed as original research (Figure 2).

### Characteristics of Pharmacogenetic Studies

We noted a marked increase in the number of primary pharmacogenetic research studies (and other types of article) since 1990 (Figure 2). The majority of articles reporting original research investigated variation in the germline (1327, 79.6%, Table 1) and of these, the greater proportion studied genetic variation in drug targets (pharmacodynamic studies; 804, 60.6%) rather than genes encoding proteins involved in drug handling and elimination (pharmacokinetic). Most pharmacogenetic studies were prospective in design (1496, 89.7%) with about one-half (852; 51.1%) set in Europe or Australasia and one-third in North America (511 studies; 30.7%). The most frequently investigated disease areas were cancer (456 studies; 27.3%), neurology/psychiatry (321 studies; 19.2%) and cardiovascular disease (287 studies; 17.2%) with a relative paucity of studies in infectious disease (106 studies, 6.4%) and respiratory medicine (49 studies, 2.9%). Most studies evaluated the intended effects of the drug under investigation (1190 studies; 71.6%); only one-eighth of studies (210, 12.6%) examined adverse drug effects, with pharmacokinetic rather than pharmacodynamic studies being more likely to do so (p = 2.02×10^−14^).

**Table 1:** Characteristics of pharmacogenetic studies included in the systematic review.

		All (n = 1668†)	Somatic (n = 341)	Dynamic (n = 804)	Kinetic (n = 465)	Full-text (n = 161)
**Sample size** Median (IQR) ‡		93 (40–222)	90 (39–281)	102 (51–273)	70 (25–136)	95 (43–246)
**Full-text articles**		161	29 (18.0%)	80 (49.7%)	48 (29.8%)	
**Study Design**, no. (%) *						
	Prospective	1,496 (89.7%)	321 (94.1%)	716 (89.1%)	411 (88.6%)	146 (90.7%)
	Case-control	92 (5.5%)	6 (1.8%)	55 (6.8%)	27 (5.8%)	9 (5.6%)
	Cross-sectional	40 (2.4%)	2 (0.6%)	20 (2.5%)	13 (2.8%)	4 (2.5%)
	Meta-analyses	32 (1.9%)	11 (3.2%)	10 (1.2%)	10 (2.2%)	1 (0.6%)
	Unable	7 (0.4%)	1 (0.3%)	3 (0.4%)	3 (0.6%)	1 (0.6%)
**Language**, no. (%)						
	English	1,638 (98.2%)	339 (99.4%)	784 (97.5%)	458 (98.5%)	161
	Other	30 (1.8%)	2 (0.6%)	20 (2.5%)	7 (1.5%)	0
**Continent of correspondence**, no. (%)						
	Europe & Australasia	852 (51.1%)	160 (46.9%)	431 (53.7%)	234 (50.3%)	84 (52.2%)
	N America	511 (30.7%)	145 (42.5%)	240 (29.9%)	105 (22.6%)	49 (30.4%)
	Asia	242 (14.5%)	27 (7.9%)	96 (12.0%)	113 (24.3%)	25 (15.5%)
	Other	55 (3.3%)	8 (2.3%)	31 (3.9%)	12 (2.6%)	3 (1.9%)
	Not specified	7 (0.4%)	1 (0.3%)	5 (0.6%)	1 (0.2%)	0
**Disease category**, no. (%)						
	Cancer	456 (27.3%)	341 (100%)	37 (4.6%)	68 (14.6%)	37 (23.0%)
	Neurology/Psychiatry	321 (19.2%)	0	228 (28.4%)	81 (17.4%)	26 (16.1%)
	Cardiovascular	287 (17.2%)	0	193 (24.0%)	76 (16.3%)	34 (21.1%)
	Endocrine	164 (9.8%)	0	135 (16.8%)	25 (5.4%)	16 (9.9%)
	Other	440 (26.4%)	0	211 (26.2%)	215 (46.2%)	48 (29.8%)
**Pharmacogenetic design**, no. (%)						
	Primary	1,364 (81.8%)	249 (73.0%)	661 (82.2%)	403 (86.7%)	137 (85.1%)
	Secondary	258 (15.5%)	77 (22.6%)	122 (15.2%)	54 (11.6%)	23 (14.3%)
	Unable	46 (2.8%)	15 (4.4%)	21 (2.6%)	8 (1.7%)	1 (0.6%)
**Outcome**, no. (%)						
	Intended effect	1,190 (71.6%)	314 (92.4%)	634 (79.2%)	208 (44.8%)	115 (71.4%)
	Adverse effect	210 (12.6%)	2 (0.6%)	116 (14.5%)	80 (17.2%)	19 (11.8%)
	Both	99 (6.0%)	17 (5.0%)	26 (3.2%)	47 (10.1%)	7 (4.3%)
	Other	164 (9.9%)	7 (2.1%)	25 (3.1%)	129 (27.8%) §	20(12.4%)
**Impact factor**, no. (%)						
	0 to 4.99**	903 (54.1%)	141 (41.3%)	453 (56.3%)	275 (59.1%)	72 (44.7%)
	5 to 9.99	507 (30.4%)	73 (21.4%)	256 (31.8%)	163 (35.1%)	59 (36.6%)
	≥10	258 (15.5%)	127 (37.2%)	95 (11.8%)	27 (5.8%)	30 (18.6%)
**P value category**, no. (%)⊥						
	Only p values<0.05	608 (74.5%)	120 (67.4%)	286 (75.7%)	177 (77.3%)	57 (70.4%)
	Mixed p values	132 (16.2%)	37 (20.8%)	52 (13.8%)	40 (17.5%)	17 (21.0%)
	Only p values>0.05	76 (9.3%)	21 (11.8%)	40 (10.6%)	12 (5.2%)	7 (8.6%)

† 58 of 1,668 studies are mixed dynamic/kinetic.

‡ Meta-analyses excluded, values are for 1504 abstracts that report a sample size. 4 of 161 full-text articles were mixed pharmacokinetic/pharmacodynamic.

*Percentages are of total.

**Includes journal that are not listed with ISI.

⊥For 816 abstracts reporting a p value or 81 full-text papers.

§ Studies investigating plasma levels of drugs.

### Genes Investigated and Number of Participants

**Figure 3:**
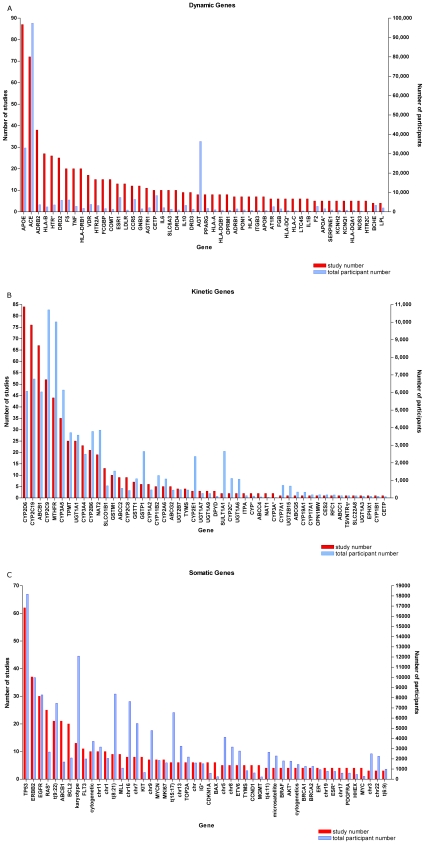
The 50 most frequently studied genes and the aggregate number of participants per gene. (a) pharmacodynamic genes (n = 305); (b) pharmacokinetic genes (n = 70); and (c) somatic genes (n = 176). * refers to >1 gene and/or non-HUGO nomenclature.

The breadth of work and the foci of activity are illustrated by the total number of genes in each category and those most frequently studied (Figure 3). There were in total 541 genes studied (176 somatic, 305 pharmacodynamic and 70 pharmacokinetic with some overlap for 10 genes). Seven genes included studies involving over 10,000 participants in aggregate: two somatic (*TP53* and non-specified karyotype mutations), 2 pharmacokinetic (*MTHFR* and *CYP2C9*) and 3 pharmacodynamic genes (*ACE*, *AGT* and *APOE*). About one-third (37.7%) of study participants were distributed among the 10 most frequently studied somatic genes; with the equivalent numbers in kinetic and dynamic studies being 68.5% and 41.8%, respectively. Thirteen of 70 (18.6%) kinetic genes, 22 of 305 (7.2%) dynamic genes and 12 of 176 (6.8%) somatic genes included more than 10 studies.

### Most Frequently Studied Gene-Drug Combinations

**Figure 4:**
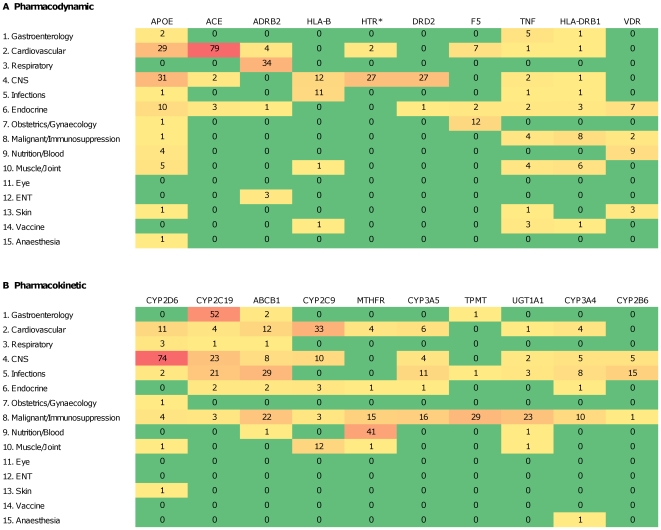
Categories of drugs evaluated in pharmacogenetic studies of the 10 most frequently studied genes. (a) pharmacodynamic; and (b) pharmacokinetic. Numbers represent total studies per gene and drug category, with cell color shading to emphasize value (heat matrix). CNS = central nervous system; ENT = ears, nose and throat. Drugs are classified as in British National Formulary (http://www.bnf.org).

The 10 most studied cancer cell gene variants were *TP53* and cisplatin/5-fluorouracil/paclitaxel response, *ERBB2* (*HER2/neu*) and anthracyclines/trastuzumab response, *EGFR* and gefitinib response, and *RAS*, *FLT3*, *ABCB1*, *BCL2* and *t(9;22)* and other karyotype and cytogenetic mutations and response to a variety of combination chemotherapy regimens. The most studied germline pharmacokinetic and pharmacodynamic genes (Figure 4) were *ACE* and cardiovascular drug response (n = 79), *CYP2D6* and response to antidepressant therapy (n = 74), *CYP2C19* and response to gastrointestinal drugs (mostly proton pump inhibitors, n = 52), *MTHFR* and response to nutritional drugs (predominantly folate, n = 41), *ADRB2* and response to respiratory medications (n = 34), *CYP2C9* and cardiovascular drugs (mainly warfarin, n = 33), *APOE* and response to drugs targeting the cardiovascular (n = 29) and central nervous system (CNS, n = 31), *TPMT* and response to chemotherapy/immunosuppression (mostly azathioprine, n = 29), and HTR* (n = 27) and *DRD2* (n = 27) and response to CNS drugs. However with the exception of *ERBB2(HER2/neu)*/trastuzumab therapy, *CYP2C9*/warfarin and *TPMT*/azathioprine none of these genes are mandated or recommended by the FDA for pharmacogenetic testing [10].

### Outcomes

We next focused on indices of clinical relevance and study quality. As in clinical trials, continuous outcome measures in pharmacogenetic studies are more likely to be surrogates for more clinically relevant binary outcomes. For example, the international normalized ratio (INR), an index of the anticoagulant effect of warfarin, might be used as a surrogate for the risk of a major hemorrhage, a serious adverse clinical event arising from warfarin treatment. From the representative subset of 161 full-text articles, continuous outcomes were more frequently reported than binary outcomes. Of a total of 546 reported outcomes, less than one-third (167, 30.6%) were binary, and these were more likely to be reported in studies of genetic variation in cancer cells (median binary outcomes/paper: 2, IQR 1–3.25) than germ-line studies (median binary outcomes/paper: 0, IQR 0-1).

### Sample Size

**Figure 5:**
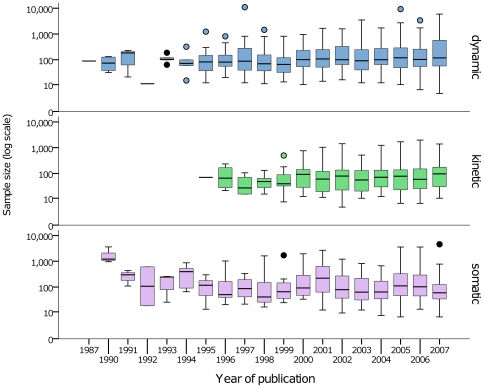
Sample size of pharmacogenetic studies from 1987 to 2007 (inclusive). Horizontal bars designate the median, boxes indicate 25^th^ and 75^th^ centiles of the distribution and vertical bars represent the non-outlier range.

Sample size in genetic studies can serve as an index of the quality and reliability because unless effect sizes are large, small studies may be inadequately powered to detect plausible genetic effects reliably [22], [23], [24]. Common alleles (those with a minor allele frequency, MAF, >0.05) tend to exert smaller effects on *disease risk* than rare alleles [25], with effect sizes for binary outcomes in gene-disease association studies being odds ratios for disease risk in the range of 1.28–1.65 [24]. Moreover, where a positive effect is seen in a small study of common alleles, a false positive association may be as or more likely than a true positive [22], [24]. In the representative subset of full text articles of pharmacogenetic research, the median MAF of the variants studied was 0.12 (IQR 0.08–0.67), suggesting that similar effect sizes for binary outcomes might be expected in pharmacogenetic studies; reliable detection of effect sizes in this range would require sample sizes in the region of 3,500 [24]. However, the vast majority of pharmacogenetic studies were far smaller (median sample size 93) and the distribution highly skewed (IQR 40–222). Moreover, there was little evidence for an increase in sample size over time (Figure 5). Although pharmacodynamic studies (median sample size 102, IQR 51–273) tended to be larger than pharmacokinetic studies (median sample size 70, IQR 25-136, p = 7.61×10^−15^) in neither case was the size of studies comparable to recent candidate gene or genome wide disease association studies [26]. Larger studies tended to achieve publication in higher than intermediate or lower impact journals (p = 2.99×10^−7^) and articles from North America, Europe & Australasia had larger sample sizes than those from Asia (p = 2.21×10^−6^). However, most articles were published in journals of modest impact factor (median 4.77, IQR 2.83–8.07; 54.1% were published in journals of impact factor <5), with no clear trend for an emergence of a larger proportion of high impact factor articles over time (p = 0.861). Impact factors were higher in studies of genetic variation in cancer cells (p = 2.07×10^−14^) and articles from North America (p = 2.17×10^−13^) compared to others in their respective groups.

### Reporting of Statistical Significance

Significance chasing bias, evidenced by a disproportionate reporting of extreme p values in small studies, previously affected candidate gene disease association studies [27]. To assess whether this might also be the case in the pharmacogenetic literature, we evaluated the distribution of reported p values in abstracts of primary research articles. About one half of study abstracts (816, 48.9%) reported a p value. Three quarters of these articles reported only significant p values (608 abstracts, 74.5%). There was no difference (p = 0.926) in the size of studies among the three p value categories: median sample size (IQR) of articles reporting only non-significant p values was 99 (57–292); mixed (significant and non-significant) p values was 103 (48–252); and only significant p values was 106 (49–252). These findings were corroborated in the detailed analysis of 161 full papers (p = 0.608).

**Figure 6:**
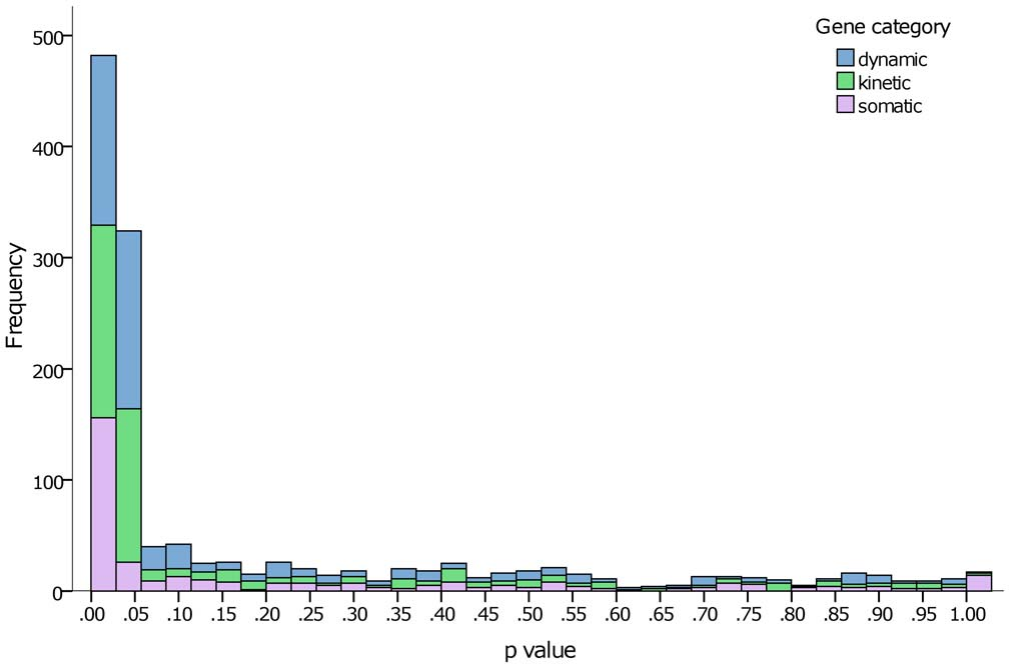
Distribution of p values in 161 full-text primary research articles in pharmacogenetics.

**Figure 7:**
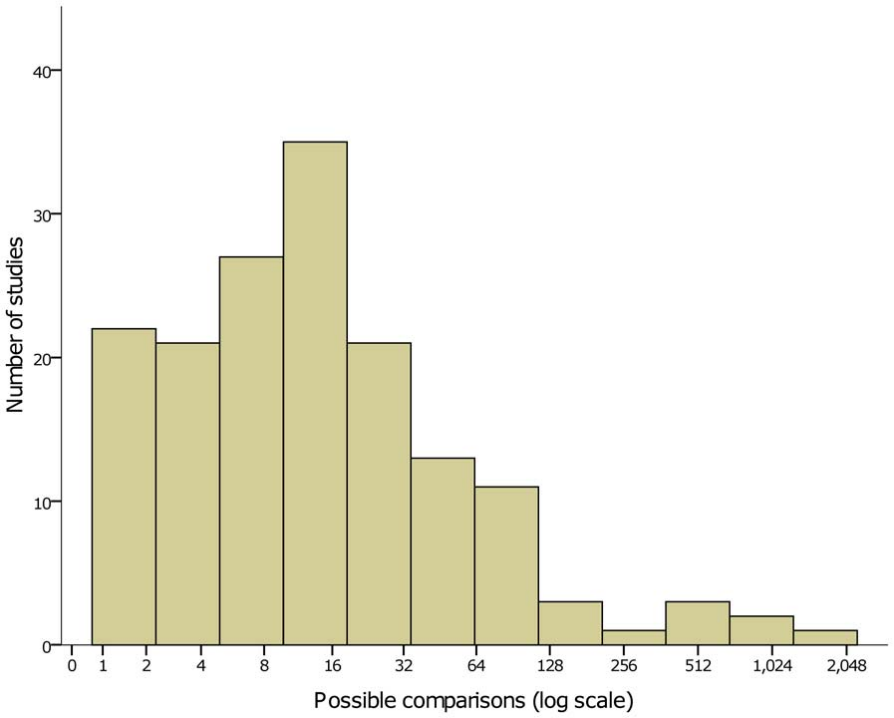
The theoretical number of total comparisons in 161 full-text articles. Calculated by multiplying the number of gene alleles studied by the number of drugs investigated by the number of outcomes recorded.

The predominance of significant p values suggests either that the prior odds of success in pharmacogenetics is higher than in most other fields of biomedical research, or that the published literature is affected by chance findings and/or publication bias [22]. Another index of significance chasing bias is the total number of hypotheses tested by any study. One hundred and twenty five of 161 full-text articles reported a p value (Figure 6) with a median of 6 p values per article (IQR 3–12). These 161 articles had a theoretical median of 12 total reportable comparisons per study (IQR 4–29, Figure 7, calculated by number of alleles x number of drugs x number of outcomes recorded), suggesting that the potential for post-hoc subgroup analysis is large in pharmacogenetic research.

### Use of Meta-Analysis

Meta-analysis has been used to strengthen conclusions regarding genetic effects on disease outcomes [28], [29], [30]. Thirty one meta-analyses of pharmacogenetic studies were identified spanning 29 genes (Table 2), 23 of which included 4 or more studies. However, a further 107 genes that were the subject of ≥4 studies had never been the subject of a meta-analysis. The majority of meta-analyses investigated variants in the germline (n = 19) with over half (n = 21) investigating intended effects and less than one-quarter (n = 7) adverse outcomes. For those genes exposed to meta-analysis, the median number of studies per gene was 22 (IQR 5–52). Six of the 7 meta-analyses in the somatic gene category (85.7%) involved the 10 most frequently studied genes, and 5 of 7 (71.4%) in the pharmacokinetic category. However, only 4 of 15 (26.7%) meta-analyses in the pharmacodynamic category involved the 10 most studied genes.

**Table 2:** Summary of meta-analyses of pharmacogenetic studies.

Gene category	Gene or region (number of meta-analyses if >1)	Total number of individual studies	Median no of studies per gene per category	Median sample size of meta-analyses (IQR) per gene per category
**Somatic** (12 meta-analyses, 7 genes)			30	503 (230–756)
	ABCB1 §	21		
	chr8	5		
	EGFR (4) §	30		
	ERBB2 (2) §	37		
	FLT3 §	11		
	RAS* §	25		
	TP53 (2) §	62		
**Dynamic** (10 meta-analyses, 15 genes)			5	2,183 (751–6,638)
	ACE §	72		
	APOA*	5		
	APOE §	87		
	CETP	10		
	ESR*	4		
	F2	5		
	F5 §	20		
	HLA*	7		
	HTR2A	15		
	HTR2C	5		
	ITPA	1		
	MKRN2	2		
	NQO2	1		
	SLC6A4	3		
	TNF §	20		
**Kinetic** (9 meta-analyses, 7 genes)			52	1,450 (161–3,029)
	ABCB1 §	67		
	CYP*	2		
	CYP2C19 §	76		
	CYP2C9 (3) §	52		
	CYP2D6 §	84		
	CYP3A4 §	23		
	CYP7A1	1		

§ In top 10 most frequently studied genes in relevant category.

*denotes more than one gene.

### FDA-Supported Pharmacogenetic Tests

We next assessed the evidence-base for pharmacogenetic tests listed by the FDA. At the time this study was performed (pre-July 09), the FDA had published guidelines on “valid genomic biomarkers”, [10] classifying pharmacogenetic tests into (i) required, (ii) recommended, and, (iii) information only. In July 2009, the website was updated [31] with removal of the classification system, however the list of “valid genomic biomarkers” and supporting references remained largely unchanged. We based our analysis on the original guidelines with accompanying classification system (Document S1).

**Figure 8:**
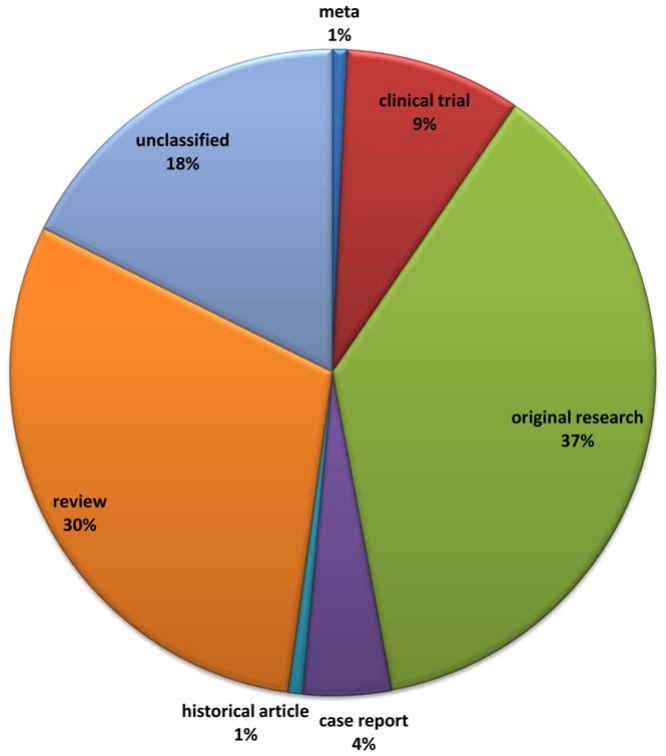
Medline annotation of studies provided by U.S. Food and Drug Administration (FDA; n = 136) as references for “approved biomarkers.”

Of the 136 references listed by the FDA in support of pharmacogenetic testing, one article was indexed in Medline as a meta-analysis (Figure 8), 63 (46%) were annotated either as clinical trials/government-supported research or comparative studies, with the remainder (48 studies, 35%) being reviews, case reports or historical articles and 24 being unclassified. Only a small proportion of the 1668 articles identified from our search mapped to relevant FDA endorsed pharmacogenetic tests (n = 101, Table S1). FDA recommended or mandated pharmacogenetic tests were more likely to investigate adverse effects, involve pharmacokinetic genes and relate to cardiovascular disease (p = 1.43×10^−16^, 1.45×10^−7^ and 5.06×10^−7^ respectively).

## Discussion

A distinctive feature of the field of pharmacogenetics is the predominance of publications indexed as reviews, commentaries, letters and other opinion based pieces over primary research articles, whichever search strategy we used to identify articles. This may have contributed to a high expectation of the delivery of personalized medicines [5], [6], [7] with modest realisation of this goal thus far. Though expanding in general, pharmacogenetic research currently centres mainly in cancer, cardiovascular and neurological/psychiatric disease with most studies being set in Europe and North America, presumably mainly among subjects of European ancestry. The relative dearth of research in other therapeutic areas (e.g. communicable disease) and among individuals of non-European ancestry, among whom there is a considerable global disease burden, may be creating an imbalance that will require addressing in future work. Even if the relevant genetic variants and effect sizes are homogeneous across different ancestral groups [32], differences in allele frequency can vary greatly [33] and such variation means that the population impact of genetic variants influencing drug response will often differ by ethnicity even if effect sizes are similar.

The major goal of pharmacogenetic research is development of genotype-based predictive tests of efficacy or toxicity. However, a prerequisite is the reliable identification of the relevant genetic loci. In genetic work, where many hundreds of thousands of hypotheses can be tested, research designs are needed that optimise the detection of true positive (while limiting the potential for false positive) association [17], [18], [19]. Despite some high quality studies, in broad terms, there are several features of the field as a whole that suggest that only a proportion of the positive associations reported are genuine. These include: the small size of most studies coupled with the more frequent evaluation of common rather than rare variants (whose effect sizes would be predicted to be small and which therefore requires large sample sizes for their reliable detection); use of surrogate (usually continuous) outcome measures rather than more clinically relevant binary outcomes; and subgroup analyses with multiple hypothesis testing. Our study may have been limited by analysing only the abstracts of articles satisfying inclusion criteria. However, detailed data (information unlikely to be reported in abstracts) on outcome measures (binary/continuous), gene variants and reported p values were derived from the full text of a subset of 10%, which accurately reflected the span of studies in the database.

Similar problems to those we highlight were recognised in the field of genetics of common disease a decade or so ago. What followed were efforts to systematically and comprehensively collate evidence from genetic association studies, large collaborative meta-analyses, larger primary studies, more comprehensive capture of genetic variation at any given locus, independent replication, and, most recently, whole genome association studies [26]. These developments have contributed to the discovery of many secure genetic associations that are providing new insights into disease pathogenesis, potential therapeutic targets and the possibility of developing predictive tests for disease. Several important and laudable efforts to collate and curate information on the genetic basis of drug response already exist, including those of the Pharmacogenetics Research Network [34]. However, the challenge in identifying primary pharmacogenetic studies is illustrated by our two alternative search strategies. Our comprehensive Medline search was sensitive (yielding >100,000 articles) but non-specific, with a large number of evaluated articles not satisfying our definition of a pharmacogenetic study. However, using a specific search strategy (via the MeSH tool) the majority of articles were missed. We know of no previous attempts to systematically identify all published pharmacogenetic studies in this way but our current analysis suggests that future attempts to do so should adopt an explicit, systematic and comprehensive search strategy such as the one we have used here. The terms “pharmacogenomic” and “pharmacogenetic” have both been used somewhat interchangeably in the literature. For example the Pharmacogenomics Knowledge database (PharmGKB; http://www.pharmgkb.org/resources/forGeneralUsers/pharmacogenetics_pharmacogenomics_and_personalized_medicine.jsp accessed 2009 November 10, archived URL http://www.webcitation.org/5lBBtDJPf) defines pharmacogenetics as “the study of … varying responses to drugs and the determination of the genetic mutations underlying these variations” and pharmacogenomics as “the study of drug response in the context of the entire genome”. However, the Human Genome Project information portal (http://www.ornl.gov/sci/techresources/Human_Genome/medicine/pharma.shtml#whatis accessed 2009 November 10, archived URL http://www.webcitation.org/5lBCB8i5T) defines pharmacogenomics as “the study of how an individual's genetic inheritance affects the body's response to drugs”. These indistinct classifications are exemplified by the U.S. National Library of Medicine's ‘controlled vocabulary’ for indexing articles via MeSH terminology: “pharmacogenomics” is not a MeSH term, on entering it in Medline, all articles indexed with the MeSH term “pharmacogenetics” are displayed.

Other developments that may be helpful include: a greater use of meta-analysis, particularly where four or more independent studies of the same gene have been conducted, perhaps with an online, continuously updated database similar to those established for Alzheimer's Disease, Parkinson's Disease and Schizophrenia [35], [36], [37], [38]. Other improvements might include: primary studies with larger sample sizes; wider use of haplotype tagging single nucleotide polymorphisms (SNPs); studies of rare and structural genetic variants whose effects are predicted to be larger, and which may therefore be more suited for use as predictive tests; and a greater focus on genes influencing drug handling and adverse effects, to fill gaps in knowledge [39].

Important studies with some of these features have been reported since the deadline we set for our literature search. For example, the identification of a SNP in the *SLCO1B1* gene, encoding the organic anion-transporting polypeptide OATP1B1, as a susceptibility factor for statin-induced myopathy involved a *genome-wide association analysis* of 85 individuals with definite or incipient statin myopathy (and 90 controls) from a trial involving over 12,000 subjects [40]. Here, the small size of the genome-wide association study belies the large-scale effort to identify the few subjects who suffer extreme adverse effects. This study provides a paradigm for the identification of genetic loci underlying rare but serious adverse effects of a commonly used drug. Other examples which could be studied in a similar way include heparin-induced thrombocytopaenia (frequency 0.5–2%), oesteonecrosis of the jaw from bisphosphonate treatment (prevalence 4–7% in those receiving intravenous bisphosphonates for hypercalcaemia of malignancy), and angio-oedema from angiotensin converting enzyme inhibitors. Because of the large genetic effect sizes that might be detected with this approach (for example an odds ratio of 17 for statin myopathy in *SLCO1B1* CC homozygotes), predictive tests may be more likely to emerge, though the rarity of the adverse effect means that rigorous assessment of the cost-effectiveness of the approach would first be required. Larger scale candidate gene studies[41], [42], [43], [44] are also providing much more secure evidence on loci influencing both drug response and adverse effects that might form the basis of predictive testing for dose adjustment or avoidance of toxic treatments.

As more reliable information begins to emerge on alleles influencing drug response from larger, better designed whole genome and candidate gene studies, focus will need to shift to the critical evaluation of the predictive performance of genetic tests in clinical practice, including studies of cost-effectiveness. These evaluations will require use of different metrics to those conventionally reported in discovery-based genetic studies (such as odds ratios or proportion of variance explained) [45], [46], [47]. Instead, sensitivity and specificity, predictive values and the generation of multivariate models that include genotype will need evaluating [42], [48]. In some cases, the most robust evaluation of the effectiveness of genetic tests may need to come from randomised trials comparing health outcomes among people randomised to pharmacogenetic testing or no testing, together with cost-effectiveness analyses as are now common when evaluating the usefulness of interventions. In concert, these efforts should help realise the promise of personalised medicines with resultant improvements in healthcare. Our recommendations for pharmacogenetic research are summarised below.

### Recommendations for Future Research in Pharmacogenetics

Primary research in pharmacogenetics should:

- give due emphasis both to adverse as well as intended effects of drugs
- be appropriately powered
- examine clinically-relevant end-points
- be conducted among individuals of non-European as well as European ancestry
- include studies of currently neglected drugs and disease areas
- enhance the likelihood of identification of large effect sizes necessary for the generation of usefully predictive tests through the study of rare or structural genetic variants, and/or more extreme phenotypic differences in response or toxicity
- ensure comprehensive SNP typing where candidate loci are studied
- utilise whole genome analysis where mechanisms are uncertain
- avoid post-hoc subgroup analysis, except where justified and powered, and report the findings with due caution
- include evidence of independent replication
- exploit existing large randomised controlled trial datasets as a resource for pharmacogenetic evaluation (e.g. *SLCO1B1* variants and statin-induced myopathy, based on the SEARCH trial involving 12,064 participants) [40]

Mechanisms should exist for:

- encouraging reporting null findings from high-quality studies
- systematically and comprehensively collating, archiving and disseminating reports of pharmacogenetic research, to highlight continuing gaps in knowledge and promote successes
- encouraging high quality updated systematic reviews and meta-analyses of pharmacogenetic research

Promising genotype-based predictive tests emerging from primary research should be:

- re-evaluated in independent prospective studies
- assessed against clinically relevant outcomes
- evaluated using the appropriate metrics for diagnostic, screening and predictive tests
- tested where appropriate in randomised trials

## Supporting Information

Document S1 U.S. Food and Drug Administration (FDA) Table of Valid Genomic Biomarkers in the Context of Approved Drug Labels (website pre-July 2009)
(3.01 MB PDF)

Table S1 U.S. Food and Drug Administration (FDA) mandated or recommended pharmacogenetic tests pre-July 2009
(0.06 MB DOC)
