# Supplementary material for: Fulfilling the Promise of Personalized Medicine? Systematic Review and Field Synopsis of Pharmacogenetic Studies
Source: PLoS One. 2009 Dec 2;4(12):e7960. doi: 10.1371/journal.pone.0007960 (PMC2778625; doi:10.1371/journal.pone.0007960)
Supplement: Document S1 — U.S. Food and Drug Administration (FDA) Table of Valid Genomic Biomarkers in the Context of Approved Drug Labels (website pre-July 2009) (3.01 MB PDF) [file pone.0007960.s001.pdf]

Table of Valid Genomic Biomarkers in the Context of Approved Drug Labels

Pharmacogenomic information is contained in about ten percent of labels for drugs approved by the FDA. A significant increase of labels containing such information has been observed over the last decade. In order to provide a reference for genomic biomarkers in labels of FDA-approved drug products, we created the table shown below. Genomic biomarkers can play an important role in identifying responders and non-responders, avoiding toxicity and adjusting the dosage of drugs to optimize their efficacy and safety. In the context of drug labels, these genomic biomarkers can be classified on the basis of their specific use, for example:

- Clinical response and differentiation,
- Risk identification,
- Dose selection guidance,
- Susceptibility, resistance and differential disease diagnosis,
- Polymorphic drug targets.

The table portrays a view on valid genomic biomarkers in the context of FDA-approved drug labels. It provides a comprehensive list of these markers and links to pharmacogenomic data, taking into account multiple regulatory contexts in which these biomarkers were approved. Most drug labels in this table provide pharmacogenomic information with no immediate recommendation for a specific action (i.e. genetic testing); however a few labels recommend or require genetic testing thereby specifying the use of these markers for reaching a therapeutic decision.

The table includes:

- Context-specific biomarker (column 1)
- Reference drug label information about the biomarker context within which the drug was approved (column 2 subsection 1)
- Test criteria (column 2 subsection 2)
- Prototypic drug associated with the label information defining the biomarker context (column 2 subsection 3)
- Other drugs in a similar context (column 3)
- Pertinent references (column 4).

Drugs sharing the context of a specific biomarker in their labels have had their pharmacogenomic information extracted into this table. This information can be accessed by placing the mouse over the symbol under the right side of the drug name. All approved drugs in this table are linked to labels at Drugs@FDA which can be accessed by clicking over symbols under the left side of the drug name. The table will be updated on a quarterly basis.

The information provided in “label context” is taken from different sections of the actual drug labels.

The term “valid” biomarker has been defined in the “Guidance for Industry: Pharmacogenomic Data Submissions”. Therein, a valid biomarker is described as a “biomarker that is measured in an analytical test system with well established performance characteristics and for which there is an established scientific framework or body of evidence that elucidates the physiologic, toxicologic, pharmacologic, or clinical significance of the test results.” The classification of biomarkers is context specific.

A critical aspect of many of these drugs is the role they play in drug-drug interactions. This list does not address

drug-drug interactions. More information on drug-drug interactions, please see Drug Development and Drug Interactions.

Reference is made to the requirement of testing for the biomarker:
1 = test required;
2 = test recommended; 2* test for at risk populations
3 = information only

Biomarker	Label Context			Examples of other Drugs Associated with this Biomarker	References (PubMed ID)
	**Representative Label**	**Test**	**Drug**		
C-KIT expression	**Gastrointestinal stromal tumor *c-Kit* expression** "*In vitro*, imatinib inhibits proliferation and induces apoptosis in gastro-intestinal stromal tumor (GIST) cells, which express an activating c-kit mutation." "Gleevec is also indicated for the treatment of patients with Kit (CD117) positive unresectable and/or metastatic malignant gastrointestinal stromal tumors (GIST)."	3	Imatinib mesylate		12851888 16226710 16294026
CCR5 -Chemokine C-C motif receptor	CCR5 is a receptor site on the human T-cell that HIV uses to bind to the cell allowing it to enter and begin replication. "SELZENTRY, in combination with other antiretroviral agents, is indicated for treatment experienced adult patients infected with only CCR5-tropic HIV-1 detectable, who have evidence of viral replication and HIV-1 strains resistant to multiple antiretroviral agents." "SELZENTRY blocks a specific receptor called CCR5 that CCR5-tropic HIV-1 uses to enter CD4 or T-cells in your blood. Your doctor will do a blood test to see if you have been infected with CCR5-tropic HIV-1 before prescribing SELZENTRY for you." "**Pharmacogenomics**	1	Maraviroc		17933726 17933725 17933724 17933723 17933722

	The impact of CCR5 promoter and coding sequence polymorphisms on the efficacy of maraviroc is being evaluated.”				
CYP2C19 Variants	**CYP2C19 Variants (Poor Metabolizers-PM and Extensive Metabolizers-EM) with genetic defect leads to change in drug exposure**. “*In vivo* studies indicated that CYP2C19 is significantly involved in the metabolism of voriconazole. This enzyme exhibits genetic polymorphism. For example, 15-20% of Asian populations may be expected to be poor metabolizers. For Caucasians and Blacks, the prevalence of poor metabolizers is 3-5%. Studies conducted in Caucasian and Japanese healthy subjects have shown that poor metabolizers have, on average, 4-fold higher voriconazole exposure (AUCτ) than their homozygous extensive metabolizer counterparts. Subjects who are heterozygous extensive metabolizers have, on average, 2-fold higher voriconazole exposure than their homozygous extensive metabolizer counterparts.”	**3**	Voriconazole	Omeprazole [m1]<br>Pantoprazole [m2]<br>Esomeprazole [m3]<br>diazepam[m4]<br>Nelfinavir [m5]<br>Rabeprazole [m6]	12867215<br>11866669
CYP2C9 Variants	**CYP2C9 Variants PM and EM genotypes and drug exposure**; “Patients who are known or suspected to be P450 2C9 poor metabolizers based on a previous history should be administered celecoxib with caution as they may have abnormally high plasma levels due to reduced metabolic clearance.”	**3**	Celecoxib		16118328<br>15637526<br>15714076<br>15037866<br>14558433
CYP2C9 Variants with Alternate Context	**CYP2C9 Variant genotypes and drug dose** “The analysis suggested an increased bleeding risk for patients carrying either the CYP2C9*2 or CYP2C9*3 alleles. Patients carrying at least one copy of	**2**	Warfarin		18034618;<br>17989110;<br>17955230

	the CYP2C9*2 allele required a mean daily warfarin dose that was 17% less than the mean daily dose for patients homozygous for the CYP2C9*1 allele. For patients carrying at least one copy of the CYP2C9*3 allele, the mean daily warfarin dose was 37% less than the mean daily dose for patients homozygous for the CYP2C9*1 allele"				
CYP2D6 Variants	**CYP2D6 Variants** "Atomoxetine is metabolized primarily through the CYP2D6 enzymatic pathway. People with reduced activity in this pathway (PMs) have higher plasma concentrations of atomoxetine compared with people with normal activity (EMs)."	**3**	Atomoxetine	Venlafaxine; [m8]<br>Risperidone; [m9]<br>Tiotropium bromide inhalation; [m10]<br>Tamoxifen; [m11]<br>Timolol Maleate; [m12]	
CYP2D6 with alternate Context	**CYP2D6 PM and EM Variants and drug exposure and risk**- "population, who are known to have a genetic defect leading to reduced levels of activity of P450 2D6. Fluoxetine, like other agents that are metabolized by P450IID6, inhibits the activity of this isoenzyme, and thus may make normal metabolizers resemble "poor metabolizers." Therapy with medications that are predominantly metabolized by the P450IID6 system and that have a relatively narrow therapeutic index should be initiated at the low end of the dose range if a patient is receiving fluoxetine concurrently or has taken it in the previous 5 weeks."	**3**	Fluoxetine HCL	Fluoxetine HCL and Olanzapine; [m13]<br>Cevimeline hydrochloride [m14]<br>Tolterodine; [m15]<br>Terbinafine; [m16]<br>Tramadol + Acetamophen [m17]<br>Clozapine [m18]<br>Aripiprazole; [m19]<br>Metoprolol; [m20]<br>Propranolol; [m21]<br>Carvedilol [m22]<br>Propafenone [m23]	16472103<br>16384813;<br>15063083;<br>16271013<br>16236141<br>15828850<br>15492763<br>15037866<br>14639062<br>10431214<br>1302039

				Thioridazine; [m24]<br>Protriptyline HCl;[m25]	
Deletion of Chromosome 5q (del(5q))	***Cytogenetic abnormality in management of Low- or Intermediate-1 risk myelodysplastic syndromes*** - “Lenalidomide is indicated for the treatment of patients with transfusion dependent anemia due to Low- or Intermediate-1-risk myelodysplastic syndromes associated with a deletion 5q cytogenetic abnormality with or without additional cytogenetic abnormalities.”	**3**	Lenalidomide		18265982<br>17893227<br>17634837<br>17379099<br>16397017
DPD Deficiency	**Deficiency of Dihydropyrimidine Dehydrogenase:** “Rarely, unexpected, severe toxicity (eg, stomatitis, diarrhea, neutropenia and neurotoxicity) associated with 5-fluorouracil has been attributed to a deficiency of dihydropyrimidine dehydrogenase (DPD) activity. A link between decreased levels of DPD and increased, potentially fatal toxic effects of 5-fluorouracil therefore cannot be excluded.	**3**	Capecitabine	Fluorouracil Cream; [m26] Fluorouracil Topical Solution & Cream[m27]	16428499<br>16163233<br>15377401<br>15093568<br>15083629
EGFR expression	**Epidermal Growth Factor Receptor presence or absence**- “EGFR expression was determined using the EGFR pharmDxTM kit. In contrast to the 1% cut-off specified in the pharmDx kit instructions, a positive EGFR expression status was defined as having at least 10% of cells staining for EGFR. The pharmDx kit has not been validated for use in pancreatic cancer. An apparently larger effect, however, was observed in two subsets: patients with EGFR positive tumors (HR = 0.68) and patients who never smoked (HR = 0.42). Analysis of the impact of EGFR	**3**	Erlotinib		16354309<br>16011858

	expression status on the treatment effect on clinical outcome is limited because EGFR status is known for 326 NSCLC study patients (45%).”				
EGFR expression with alternate Context	**Epidermal Growth Factor Receptor presence or absence**- “Pretreatment assessment for evidence of EGFR expression is not required for patients with squamous cell carcinoma of head and neck (SCCHN)”	**3**	Cetuximab Head and Neck Cancer	Gefitinib[m28]	16354869 16336755 16336752 16117976 16061873 15962524 15863375 15946581 15677699 15217966
EGFR expression with alternate Context	**Epidermal Growth Factor Receptor presence or absence**- “Patients enrolled in the clinical studies were required to have immuno-histochemical evidence of positive EGFR expression using the DakoCytomation EGFR pharmDx™ test kit.”	**1**	Cetuximab Colorectal Cancer	Panitumab [m35] Gefitinib [m28]	16354869 16336755 16336752 16117976 16061873 15962524 15863375 15946581 15677699 15217966
Familial Hypercholestremia (deficiency, and/or mutation, of receptors for low density lipoprotein -LDL)	***Dosage adjustment for Homozygous and heterozygous Familial Hypercholestremia “***Doses should be individualized according to the recommended goal of therapy. Homozygous Familial Hypercholestremia (10-80mg/day)and heterozygous (10-20mg/day) Familial Hypercholestremia dose adjustment needed in pediatric patients”	**2**	Atorvastatin		17056835 14512370 12145769
G6PD Deficiency	**G6PD deficiency and risk** “Rasburicase administered to patients with glucose-phosphate dehydrogenase (G6PD) deficiency can cause severe hemolysis. ELITK administration should be immediately and permanently discontinued in any patient developing hemolysis. It is recommended that patients at higher risk for G6PD deficiency (e.g., patients of African or Mediterranean ancestry) be screened prior to	**2**	Rasburicase	Dapsone[m29]	16204390 11842483

	starting ELITEK therapy (see CONTRANDICATIONS and WARNINGS, Hemolysis)."				
G6PD Deficiency with alternate Context	**G6PD deficiency and tolerance** "Hemolytic reactions (moderate to severe) may occur in glucose-6-phosphate dehydrogenase (G-6-PD) deficient. If primaquine phosphate is prescribed for an individual with erythrocytic glucose-6-phosphatedehydrogenase (G-6-PD) deficiency or nicotinamide adenine dinucleotide (NADH) methemoglobin reductase deficiency, the person should be observed closely for tolerance."	**3**	Primaquine	Chloroquine [m30]	15330059<br>15117307<br>12643993<br>9685977
Her2/neu Over-expression	**Overexpresion of Her2/neu necessary for selection of patients appropriate for drug therapy "**Detection of HER2 protein overexpression is necessary for selection of patients appropriate for HERCEPTIN therapy (see INDICATIONS)." "HERCEPTIN should be used in patients whose tumors have been evaluated with an assay validated to predict HER2 protein overexpression (see PRECAUTIONS: HER2 Testing and CLINICAL STUDIES: HER2 Detection)."	**1**	Trastuzumab	Lapatinib [m36]	18188694<br>17921724<br>17909820<br>16445668<br>16277882<br>16137435<br>15725114<br>15143970<br>14990641
HLA-B*1502 allele presence	"SERIOUS DERMATOLOGIC REACTIONS AND HLA-B*1502 ALLELE." "Prior to initiating Tegretol therapy, testing for HLA-B*1502 should be performed in patients with ancestry in populations in which HLA-B*1502 may be present. In deciding which patients to screen, the rates provided above for the prevalence of HLA-B*1502 may offer a rough guide, keeping in mind the limitations of these figures due to wide variability in rates	**2***	Carbamazepine		18192896<br>18085998<br>17697703<br>17620823<br>17509004<br>16981842<br>16538176<br>16415921<br>15057820

	even within ethnic groups, the difficulty in ascertaining ethnic ancestry, and the likelihood of mixed ancestry. Tegretol should not be used in patients positive for HLA-B*1502 unless the benefits clearly outweigh the risks. Tested patients who are found to be negative for the allele are thought to have a low risk of SJS/TEN (see **WARNINGS** and **PRECAUTIONS/Laboratory Tests**).” ”For genetically at-risk patients (See WARNINGS), high-resolution ‘*HLA-B*1502 typing*’ is recommended. The test is positive if either one or two HLA-B*1502 alleles are detected and negative if no HLA-B*1502 alleles are detected.”				
HLA-B*5701 allele presence	WARNING: HYPERSENSITIVITY REACTIONS/LACTIC ACIDOSIS AND SEVERE HEPATOMEGALY<br>Patients who carry the HLA-B*5701 allele are at high risk for experiencing a hypersensitivity reaction to abacavir. Prior to initiating therapy with abacavir, screening for the HLA-B*5701 allele is recommended; this approach has been found to decrease the risk of hypersensitivity reaction. Screening is also recommended prior to reinitiation of abacavir in patients of unknown HLA-B*5701 status who have previously tolerated abacavir. HLA-B*5701-negative patients may develop a suspected hypersensitivity reaction to abacavir; however, this occurs significantly less frequently than in HLA-B*5701-positive patients. Regardless of HLA-B*5701 status, permanently discontinue ZIAGEN if	**2**	Abacavir		18256392<br>18444831<br>18614879<br>18549801<br>18505179<br>18370849<br>18332899<br>18303141<br>18192781<br>18184080<br>18025891<br>18018760<br>17885623<br>17631508<br>17620824<br>17534855<br>17356469

	hypersensitivity cannot be ruled out, even when other diagnoses are possible<br>*Risk Factor: HLA-B*5701 Allele:* Studies have shown that carriage of the HLA-B*5701 allele is associated with a significantly increased risk of a hypersensitivity reaction to abacavir.<br><br>*Before starting ZIAGEN, tell your doctor about all of your medical conditions, including if you: have been tested and know whether or not you have a particular gene variation called HLA-B*5701.*				
NAT Variants	**N-acetyltransferase slow and fast acetylators and toxicity-** "slow acetylation may lead to higher blood levels of the drug, and thus, an increase in toxic reactions."	**3**	Rifampin, isoniazid, and pyrazinamide	Isosorbide dinitrate and Hydralazine hydrochloride [m31]	12669770<br>12715953<br>2224079<br>12271964<br>11259359<br>11677864<br>15951616
Philadelphia Chromosome-positive responders	**Philadelphia (Ph1) chromosome presence (effective) -**"Busulfan is clearly less effective in patients with chronic myelogenous leukemia who lack the Philadelphia (Ph**1**) chromosome. Also, the so-called "juvenile" type of chronic myelogenous leukemia, typically occurring in young children and associated with the absence of a Philadelphia chromosome, responds poorly to busulfan. The drug is of no benefit in patients whose chronic myelogenous leukemia has entered a "blastic" phase."	**3**	Busulfan		11919388
Philadelphia Chromosome-positive responders with alternate context	**Philadelphia (Ph1) chromosome presence (effective)** "Dasatinib is indicated for the treatment of adults with Philadelphia chromosome-positive acute lymphoblastic leukemia (Ph+ ALL) with resistance or intolerance to prior therapy"	**1**	Dasatinib		17461740<br>14633780

PML/RAR alpha gene expression (Retinoic acid receptor responder and non-responders)	**PML/ RAR (alpha) fusion gene presence** "Initiation of therapy with VESANOID may be based on the morphological diagnosis of acute promyelocytic leukemia (APL). Confirmation of the diagnosis of APL should be sought by detection of the t (15; 17) genetic marker by cytogenetic studies. If these are negative, PML/ RAR (alpha) fusion should be sought using molecular diagnostic techniques. The response rate of other AML subtypes to VESANOID has not been demonstrated; therefore, patients who lack the genetic marker should be considered for alternative treatment."	**3**	Tretinoin	Arsenic Oxide [m32]	15735696 11114710
Protein C deficiencies (hereditary or acquired)	**Hereditary or acquired deficiencies of protein C or its cofactor, protein S**, has been associated with tissue necrosis following warfarin administration. "Not all patients with these conditions develop necrosis, and tissue necrosis occurs in patients without these deficiencies. Inherited resistance to activated protein C has been described in many patients with venous thrombosis-embolic disorders but has not yet been evaluated as a risk factor for tissue necrosis. The risk associated with these conditions, both for recurrent thrombosis and for adverse reactions, is difficult to evaluate since it does not appear to be the same for everyone. Decisions about testing and therapy must be made on an individual basis."	**2**	Warfarin		16444443 10718793 8913415
TPMT Variants	"**Thiopurine methyltransferase deficiency or lower activity** due to mutation at increased risk of myelotoxicity. TPMT testing is recommended and consideration be given to	**2**	Azathioprine	Thioguanine [m33] Mercaptopurine [m34]	16409140 16267626 15792824 15571264 15570193 15316356 15226673

	either genotype or phenotype patients for TPMT"				14985890<br>12465143<br>10628931<br>7857117
UGT1A1 Variants	**UGT1A1 mutation in patients, exposure to drug and hence their susceptibility to toxicity.** "Individuals who are homozygous for the UGT1A*28 allele are at increased risk for neutropenia following initiation of camptosar treatment. A reduced initial dose should be considered for patients known to be homozygous for the UGT1A*28 allele. Heterozygous patients may be at increased risk of neutropenia; however clinical results have been variable and such patients have been shown to tolerate normal starting doses"	**2**	Irinotecan		15297419<br>15280927<br>15007088<br>15084617
UGT1A1 variants with alternate context	**Pharmacogenomics (safety)** "Tasigna can increase bilirubin levels. A pharmacogenetic analysis of 97 patients evaluated the polymorphisms of UGT1A1 and its potential association with hyperbilirubinemia during Tasigna treatment. In this study, the (TA)7/(TA)7 genotype was associated with a statistically significant increase in the risk of hyperbilirubinemia relative to the (TA)6/(TA)6 and (TA)6/(TA)7 genotypes. However, the largest increases in bilirubin were observed in the (TA)7/(TA)7 genotype (UGT1A1*28) patients [See Warnings and Precautions (5.5)]."	**3**	Nilotinib		17611564
Urea Cycle Disorder (UCD) Deficiency	**Urea cycle disorders** – "Prior to the initiation of valproate therapy, evaluation for UCD should be considered"	**2**	Valproic acid	Sodium Phenylacetate and Sodium Benzoate; sodium phenyl butyrate	16100994<br>16136341<br>15465784<br>10098145
Vitamin K epoxide	**Vitamin K epoxide reductase**	**2**	Warfarin		18034619;

reductase (VKORC1) Variants	**(VKOR) Variant-** ""Certain single nucleotide polymorphisms in the VKORC1 gene (especially the -1639G).A allele) have been associated with lower dose requirements for warfarin. About 55% of the variability in warfarin dose could be explained by the combination of VKORC 1 and CYP2C9 genotypes, age, height, body weight, interacting drugs, and indication for warfarin therapy in Caucasian patients. Similar observations have been reported in Asian patients."				18030307; 17955831; 17912794; 17906972

Notes

Cited by:

Frueh FW, Amur S, Mummaneni P, Epstein RS, Aubert RE, Deluca TM, Verbrugge RR, Burckart GJ, Lesko LJ.
Pharmacogenomic biomarker information in drug labels approved by the United States food and drug administration: prevalence of related drug use.
Pharmacotherapy. 2008 Aug;28(8):992-8

Nakamura Y.
Pharmacogenomics and Drug Toxicity.
N Engl J Med. 2008 Jul 23

Shashi Amur, Felix Frueh, Lawrence Lesko and Shiew-Mei Huang
Integration and use of biomarkers in drug development, regulation and clinical practice: a US regulatory perspective
Biomarkers in Medicine, June 2008; Vol. 2, No. 3, Pages 305-311 (doi:10.2217/17520363.2.3.305), http://www.futuremedicine.com/doi/abs/10.2217/17520363.2.3.305

Marrer E, Dieterle F.
Promises of biomarkers in drug development--a reality check.
Chem Biol Drug Des. 2007 Jun; 69(6):381-94

Goodsaid F, Frueh F.
Biomarker qualification pilot process at the US Food and Drug Administration.
AAPS J. 2007 Mar 23;9(1):E105-8

Back to Top   Back to Genomics

PDF requires the free Adobe Acrobat Reader

Date created: September 15, 2006, Updated September 10, 2008
