# Supplementary material for: Fulfilling the Promise of Personalized Medicine? Systematic Review and Field Synopsis of Pharmacogenetic Studies
Source: PLoS One. 2009 Dec 2;4(12):e7960. doi: 10.1371/journal.pone.0007960 (PMC2778625; doi:10.1371/journal.pone.0007960)
Supplement: Table S1 — U.S. Food and Drug Administration (FDA) mandated or recommended pharmacogenetic tests pre-July 2009 (0.06 MB DOC) [file pone.0007960.s002.doc]

**Table S1. U.S. Food and Drug Administration** **(FDA) mandated or recommended pharmacogenetic tests pre-July 2009**

| **FDA guidance** | **Gene** | **Drug** | **Relevant UK guidance** | **Current Database** | | |
| --- | --- | --- | --- | --- | --- | --- |
|  |  |  |  | **Number of relevant studies** | **Total number of partici-pants** | **Number of meta-analyses** |
| **Test required** | CCR5 | Maraviroc | Gazzard B. British HIV Association guidelines for the treatment of HIV-1 infected adults with antiretroviral therapy. HIV Medicine 2008. Co-receptor tropism should be determined prior to using maraviroc.  Available: <http://www.bhiva.org/files/file1030835.pdf> Accessed 2009 October 24. | 10 | 6,117 | 0 |
|  | *EGFR* | Cetuximab | NICE – inclusion criteria for cetuximab use: patients with EGFR-expressing metastatic colorectal cancer who have previously failed irinotecan-including therapy.  Available: <http://www.nice.org.uk/nicemedia/pdf/Final_protocol_Bowelcancer_0605.pdf> Accessed 2009 October 24. | 3 | 470 | 1 |
|  | *Her2/neu* | Trastuzumab | NICE - Trastuzumab monotherapy is recommended as an option for people with tumours expressing HER2 scored at levels of 3+ who have received at least two chemotherapy regimens for metastatic breast cancer.  HER2 levels should be scored using validated immunohistochemical techniques and in accordance with published guidelines. Licensed for two indications for the treatment of MBC overexpressing HER2 at level 3+.  Firstly, it is licensed in combination with paclitaxel for patients with MBC who have not received chemotherapy for metastatic disease and in whom an anthracycline is unsuitable.  Secondly, it is licensed as a monotherapy for patients who have received at least two chemotherapy regimens for MBC; prior chemotherapy must have included at least an anthracycline and a taxane, unless these treatments are inappropriate; patients who are oestrogen receptor-positive must also have failed to respond to appropriate hormonal therapy.  Available: <http://www.nice.org.uk/nicemedia/pdf/advancedbreastcancerno34PDF.pdf> Accessed 2009 October 24. | 4 | 1,042 | 0 |
|  | Philadel-phia chro-mosome | Dasatinib | NICE – ALL guidance suspended .  Available: <http://www.nice.org.uk/Guidance/TA/Wave17/6> Accessed 2009 October 24.  NICE – CLL guidance in progress.  Available: <http://www.nice.org.uk/Guidance/TA/Wave17/18> Accessed 2009 October 24. | 4 | 319 | 0 |
| **Test recomm-ended** | CYP2C9 | Warfarin | British Committee for Standards in Haematology - no specific comments in relation to *CYP2C9*. Available: <http://www.bcshguidelines.com/guidelinesMENU.asp> Accessed 2009 October 24. | 29 | 8,272 | 3 |
|  | Familial hyper-cholestr-aemia | Atorvastatin | NICE – “Healthcare professionals should consider prescribing a high-intensity statin to achieve a recommended reduction in LDL-C concentration of greater than 50% from baseline (that is, LDL-C concentration before treatment). FH diagnosed using a combination of DNA testing and LDL-C concentration measurement.”  Available: <http://www.nice.org.uk/guidance/index.jsp?action=download&o=41698> Accessed 2009 October 24. | 1 | 12 | 0 |
|  | G6PD deficiency | Rasburicase | NICE – *no guidance;* BNF: listed as a drug with a possible risk of haemolysis in some G6PD-deficient individuals  Available: [http://bnf.org/bnf/bnf/current/4925.htm?q=%22rasburicase%22#_hit](http://bnf.org/bnf/bnf/current/4925.htm?q="rasburicase"" \l "_hit) Accessed 2009 October 24. | 0 (FDA evidence = 2 case reports) | 0 | 0 |
|  | HLA-B*5701 allele presence | Abacavir | British HIV Association: “Kivexa should be used only in patients who are HLA-B*5701 negative” Available: <http://www.bhiva.org/files/file1030835.pdf> Accessed 2009 October 24. | 11 | 1,726 | 0 |
|  | Protein C deficiency | Warfarin | British Committee for Standards in Haematology  “There is no reason to believe that any change to the standard regimen for induction of anticoagulation is indicated in these [protein C] deficiency states.”  Available: <http://www.bcshguidelines.com/pdf/BJH512.pdf> Accessed 2009 October 24. | 2 | 246 | 0 |
|  | *TPMT* variants | Azathioprine | Drugs and Therapeutic Bulletin: “We believe it is a sensible precaution to test all patients starting on azathioprine for TPMT enzyme activity.”  Available: <http://dtb.bmj.com/cgi/content/full/47/1/9> Accessed 2009 October 24. | 16 | 1,173 | 0 |
|  | *UGT1A1* variants | Irinotecan | NICE guidelines – in progress (but not specifically on this question).  Available:  <http://www3.interscience.wiley.com/cgi-bin/fulltext/121382526/HTMLSTART> Accessed 2009 October 24. | 13 | 1,085 | 0 |
|  | Urea cycle disorder deficiency | Valproic acid | Unable to find any UK recommendations. NICE guidance on epilepsy.  Available: <http://www.nice.org.uk/nicemedia/pdf/CG020NICEguideline.pdf> Accessed 2009 October 24. | 0 (FDA evidence = 4 case reports) | 0 | 0 |
|  | Vitamin K epoxide reductase variant | Warfarin | No UK guidelines (NICE or British Committee for Standards in Haematology); From USA: National Guidelines Clearinghouse: “The work group feels that more clinical trials are necessary before recommending routine testing of patients for these [VKORC1 AND CYP2C9] genetic variations. There are many other variables that influence a patient's response to warfarin therapy. Most important is that all patients initiating warfarin need frequent, careful monitoring to assess their response to this therapy.”  Available:  <http://www.guideline.gov/summary/summary.aspx?doc_id=11529&nbr=5971&ss=6&xl=999> Accessed 2009 October 24. | 7 | 1,166 | 0 |
| **Test for at risk popula-tions** | HLA-B*1502 allele presence | Carbama-zepine | Drug Safety Update (MHRA): “Individuals of Han Chinese, Hong Kong Chinese, or Thai origin should be screened for the *HLA-B*1502* allele before prescription of carbamazepine because of a risk of severe skin  reactions, particularly Stevens-Johnson syndrome”  Available:  http://[www.mhra.gov.uk/home/idcplg?IdcService=GET_FILE&dDocName=CON033504&RevisionSelectionMethod=Latest](http://www.mhra.gov.uk/home/idcplg?IdcService=GET_FILE&dDocName=CON033504&RevisionSelectionMethod=Latest) Accessed 2009 October 24. | 6 | 183 | 0 |

Although the total number of FDA-matched studies in this table is 106, 5 of these are represented twice (e.g. studies investigating *CYP2C9* and *VKORC1* with warfarin) thus the actual total is 101. Pharmacogenetic studies categorised as ‘information only’ on the FDA website were not included in our analysis. Derived from original FDA classification pre-July 2009.[10]
